# Exploring New Drug Repurposing Opportunities for MEK Inhibitors in RASopathies: A Comprehensive Review of Safety, Efficacy, and Future Perspectives of Trametinib and Selumetinib

**DOI:** 10.3390/life14060731

**Published:** 2024-06-06

**Authors:** Andrea Gazzin, Federico Fornari, Simona Cardaropoli, Diana Carli, Marco Tartaglia, Giovanni Battista Ferrero, Alessandro Mussa

**Affiliations:** 1 Department of Molecular Biotechnology and Health Sciences, Molecular Biotechnology Center, University of Turin, 10126 Turin, Italy; andrea.gazzin@unito.it; 2 Clinical Pediatrics Genetics Unit, Regina Margherita Children’s Hospital, 10126 Turin, Italy; 3 Postgraduate School of Pediatrics, Department of Public Health and Pediatrics, University of Turin, 10126 Turin, Italy; 4 Department of Medical Sciences, University of Turin, 10126 Turin, Italy; 5 Molecular Genetics and Functional Genomics, Bambino Gesù Children’s Hospital IRCCS, 00165 Rome, Italy; 6 Department of Clinical and Biological Sciences, University of Turin, 10043 Orbassano, Italy

**Keywords:** RASophaty, MEK inhibitor, drug repurposing, trametinib, selumetinib

## Abstract

The RASopathies are a group of syndromes caused by genetic variants that affect the RAS-MAPK signaling pathway, which is essential for cell response to diverse stimuli. These variants functionally converge towards the overactivation of the pathway, leading to various constitutional and mosaic conditions. These syndromes show overlapping though distinct clinical presentations and share congenital heart defects, hypertrophic cardiomyopathy (HCM), and lymphatic dysplasia as major clinical features, with highly variable prevalence and severity. Available treatments have mainly been directed to target the symptoms. However, repurposing MEK inhibitors (MEKis), which were originally developed for cancer treatment, to target evolutive aspects occurring in these disorders is a promising option. Animal models have shown encouraging results in treating various RASopathy manifestations, including HCM and lymphatic abnormalities. Clinical reports have also provided first evidence supporting the effectiveness of MEKi, especially trametinib, in treating life-threatening conditions associated with these disorders. Nevertheless, despite notable improvements, there are adverse events that occur, necessitating careful monitoring. Moreover, there is evidence indicating that multiple pathways can contribute to these disorders, indicating a current need to more accurate understand of the underlying mechanism of the disease to apply an effective targeted therapy. In conclusion, while MEKi holds promise in managing life-threatening complications of RASopathies, dedicated clinical trials are required to establish standardized treatment protocols tailored to take into account the individual needs of each patient and favor a personalized treatment.

## 1. Introduction

The RASopathies are a group of syndromes caused by variants in genes encoding components of the RAS–mitogen-activated protein kinase (MAPK) signaling pathway, a key regulator of cell survival, differentiation, and proliferation. In RASopathies, enhanced MAPK signaling is generally observed due to gain-of-function or loss-of-function variants. More than 20 genes implicated in RASopathies have been identified. In Noonan syndrome (NS), the most common and clinically variable among RASopathies, variants most commonly affect the *PTPN11* (50%), *SOS1* (10%), *LZTR1* (10%), *RAF1* (5%), and *RIT1* (5%) genes; most rarely, *SOS2*, *KRAS*, *BRAF*, *MAP2K1*, *MRAS*, *NRAS*, *RRAS2*, and *SPRED2* can be involved [1,2]. Besides NS, the RASopathy family includes neurofibromatosis type 1 (NF1), NS with multiple lentigines (previously known as LEOPARD syndrome), Costello Syndrome (CS), cardio-facio-cutaneous syndrome (CFCS), Legius syndrome, Mazzanti syndrome, capillary malformation–arteriovenous malformation syndrome (CM-AVM), and other emerging clinically related disorders [3,4]. Somatic variants in a number of the same genes (e.g., *KRAS*, *HRAS* and *NRAS*) underlie mosaic RASopathies, including oculoectodermal syndrome [5], encephalo-cranio-cutaneous lipomatosis [6], Schimmelpenning–Feuerstein–Mims syndrome [7], cutaneous skeletal hypophosphatemic syndrome (CSHS) [8], and vascular malformations [9,10,11,12,13,14,15,16]. Constitutional RASopathies have significant phenotypic overlap encompassing dysmorphic facial features, congenital heart defects (CHDs) and hypertrophic cardiomyopathy (HCM), growth failure, skeletal and ectodermal abnormalities, lymphatic dysplasia, cryptorchidism, bleeding diathesis, and various degrees of neurodevelopmental delay (NDD)/intellectual disability (ID). It is evident from the clinical practice that a significant portion of patients present severe or lethal phenotypes, with progressive complications highly impacting morbidity and mortality [3,17]. HCM is a major determinant of the outcome in patients with NS [18] implying long-term morbidity [19], and high mortality rate [1]. Infants diagnosed with HCM in this context face a challenging prognosis, with a 15% mortality rate for those diagnosed within the first year of life [20]. Current treatment for HCM is based on symptomatic therapy: beta-blockers represent the first-line therapy, calcium channel blockers, disopyramide, and diuretics are often implemented, and surgical treatments (myomectomy or alcohol ablation) are options for cases with critical cardiac outflow obstruction. These interventions, however, have proven to have poor efficacy and are associated with a high risk of morbidity: orthotopic heart transplant is the only option left as an alternative [19,21,22]. Lymphatic dysplasia, a major feature of RASopathies affecting more than 20% of NS spectrum disorders patients, has a significant impact on clinical course and morbidity [23]. Therapeutic options to reduce lymphatic accumulation and leakage include thoracentesis/paracentesis, medium-chain triglyceride-based diet, octreotide, and diuretics. Treatment for severe and unresponsive lymphatic loss could include invasive surgical and interventional techniques such as ligation, embolization, or anastomosing of lymphatic vessels [24,25,26].

With this regard, the therapeutic options are aimed at obtaining symptomatic relief and do not act directly on the disrupted RAS-MAPK signaling. Sirolimus has shown benefit in treating the functional consequences of lymphatic abnormalities in a consistent number of cases, despite multiple adverse events in the presence of a response often incomplete. In addition, there is a complete lack of data from randomized controlled trials and the data available must be interpreted with caution [27,28,29].

### 1.1. Ras-MAPK Signaling as Target

**Figure 1:**
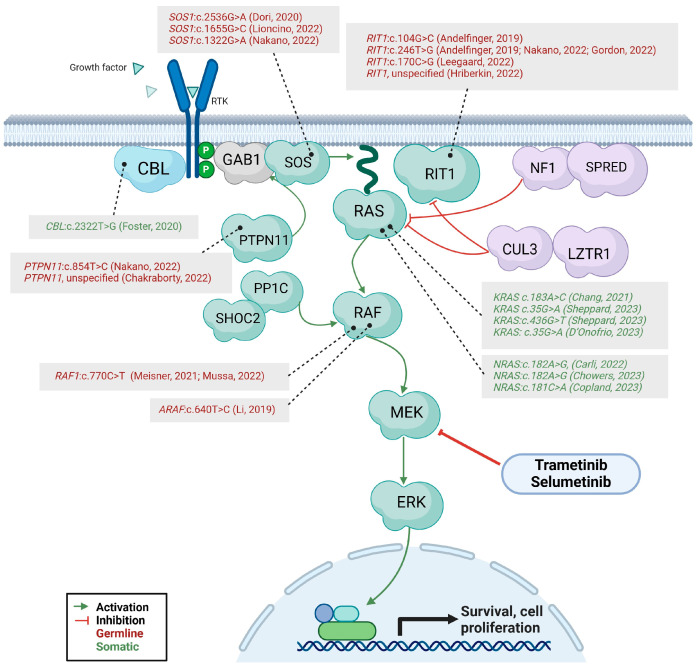
The RAS/MAPK intracellular pathway: The pathogenetic variants of patients treated with MEKi as found in the literature search are shown in their position within the intracellular pathway. The molecular targets of MEKi trametinib and selumetinib are also displayed [26,33,34,35,36,37,38,39,40,41,42,43,44,45,46,47,48].

RAS mutations are found in nearly 20% of human cancers [30] and, in approximately 30% of them, an aberrantly activated RAS-MAPK signaling is found [31]. Therefore, the Ras-MAPK pathway has represented an attractive target for cancer treatment, and multiple small molecule inhibitors directed against signal transducers operating within this signaling cascade have been developed or are currently under evaluation as cancer therapies (Figure 1). In particular, the dual-specificity protein kinases MEK1/2, crucial components of the RAS-MAPK pathway, have been the target of several inhibitors that were tested and approved for oncological indications [32]. As biochemically the molecular mechanism underpinning RASopathies corresponds to an overactivation of the RAS-MAPK cascade, the experience from oncology has provided encouraging data to extend treatment indication of the MEK inhibitors (MEKis) to the RASopathies.

### 1.2. Pre-Clinical Studies

In vitro and animal models of RASopathy, designed to test the efficacy of MEKi, showed promising results in ameliorating various aspects of RASopathies. The MEKi PD0325901 demonstrated efficacy on the treatment of HCM and other syndromic aspects in various RASopathy mouse models [49,50,51,52]. The same MEKi showed functional normalization of the sarcomere structures and contraction kinetics of myocardium in the in vitro model of cardiomyocyte derived from the RAF1^S257L^-iPSC line [53]. Lymphatic dysplasia also showed improvements under MEKi in dermal lymphatic endothelial cell organoids and zebrafish models of Kras^G12D^ and Kras^G13D^ variants, significantly reducing lymphatic dysplasia [33]. Finally, encouraging results were also obtained in rescuing the myopathy phenotype [51,54], in ameliorating body development defects [55], and normalizing cognitive impairment [56] in several animal models.

Taken together, these experiments provided valuable insights into the treatment of clinically relevant evolutive manifestations of the RASopathies, leading to the idea of using MEKi in these patients for treating life-threatening complications of these disorders where no other treatment options are available.

It should be noted that non-tumoral use of the Ras-MAPK inhibitor has not been approved yet [57] and clinical trial are lacking despite reports of the off-label treatment results accumulating in the medical literature. Among the several drugs modulating the RAS/MAPK signal, MEKi showed the most interesting results in this field. Among the orally available MEKi (i.e., selumetinib, trametinib, binimetinib, cobimetinib and mirdametinib), trametinib is the molecule that has been most frequently elected for such conditions and for which results have been most extensively published [26,33,34,35,36,37,38,39,40,41,42,43,44,45,46,47,58,59]. Selumetinib administration in RASopathy was reported once [35]. No other MEKiuse in humans has been reported in the literature, with trametinib and selumetinib being the only two molecules reported so far. This review summarizes such publications to provide a comprehensive understanding of the clinical indications, therapeutic effects, adverse events, and perspectives of the use of trametinib to treat various manifestations in RASopathies.

## 2. Methods

A literature search was conducted selecting reports of patients affected by RASopathies and treated with MEKi. Data from the literature were collected through searches in PubMed and manuscripts were screened to gather references that were missing after the first selection. The following search string was used: (“Noonan syndrome” OR “costello” OR “leopard” OR “capillary malformation arteriovenous malformation syndrome” OR “cardiofaciocutaneous” OR “legius” OR “rasopathy” OR “rasopathies”) AND (“cobimetinib” OR “L01EE02” OR “GDC-0973” OR “selumetinib” OR “AZD6244” OR “ARRY-142886” OR “binimetinib” OR “MEK162” OR “ARRY-162” OR “ARRY-438162” OR “trametinib” OR “MEK inhibitor” OR “GSK1120212”). Duplicates were excluded and the remaining articles were filtered based on title and abstract, excluding those related to preclinical studies, related to tumors, and those available in languages other than English. Finally, articles not providing any information about trametinib treatment in humans or not reporting the results of the treatment were excluded after full text evaluation. The literature search was last updated on 29 February 2024. AG and AM reviewed each report independently. The data sought encompassed genotype, age at therapy start, dose administered (mg/kg/day or mg/kg), route of administration, time first clinical change noticed, therapy administration period, observation period, clinical indication for which therapy was started, therapeutic effect, side effects, grade of side effect, time at follow-up, and exitus.

## 3. Literature Search Results

Nineteen papers were finally included, providing data on a total of 24 patients (Table 1). Twenty-three patients were treated with trametinib and one patient was treated with selumetinib.

### 3.1. Cardiological Use of MEKi

In 2019, Andelfinger and colleagues reported the results of the first administration of trametinib in two children of 13 and 14 weeks of age with HCM and NS due to *RIT1* variants. After three months of treatment with an oral dose ranging from 0.020 to 0.030 mg/kg/day, both patients showed a prompt and dramatic clinical improvement with nt-pro-BNP normalization and HCM regression. Such effects were stable over 17 months of treatment and both patients showed improved growth after treatment initiation. No adverse event was reported. The researchers hypothesized that MEK inhibition may be more effective during a fixed time window before the onset of cardiac remodeling [34]. In 2022, Mussa and colleagues also administered 0.022 mg/kg/day of trametinib to a neonate affected by NS and the *RAF1* pathogenic variant with a severe HCM, reporting similar effectiveness. An immediate decrease in nt-pro-BNP and cardiac failure score was promptly followed by improvement in clinical condition, enabling weaning from inotropes and mechanical ventilation. Indeed, cardiac US showed a tendency towards HCM improvement as well as a reduction in the septal thickness from treatment start. However, despite improvement in myocardial thickness and function, the severe pulmonary hypertension due to microvessel malformation and rapid pulmonary artery dilatation worsened following neurosurgery for intraventricular hemorrhage and ultimately lead this patient to death at 57th day of life [58]. Leegard and colleagues also used trametinib to treat a 6-month toddler with the *RIT1* variant and HCM. From an initial dose of 0.025 mg/kg/day, they escalated the dose depending on weight gain and response to 0.040 mg/kg/day. After one month of treatment, HCM regressed by 30%, and after 21 months of treatment HCM was completely regressed. A significant reduction in pulmonary valve stenosis was also reported. The authors reported mild adverse effects involving the skin [43].

Trametinib was reported to be successful in treating two patients with HCM and incessant multifocal atrial tachycardia (MAT). Lioncino and colleagues described a 9-week-old toddler with the *SOS1* variant with untreatable pleural effusion and MAT with heart failure successfully treated with oral trametinib administration at 0.020 mg/kg/day; they documented a dramatic clinical change after three days with resolution of the pleural effusion, anti-arrhythmic drugs withdrawal, and rapid improvement in respiratory parameters enabling mechanical ventilation weaning [45]. Similarly, Meisner and colleagues treated a patient with severe MAT and heart failure, obtaining a reduction in the frequency of arrhythmic episodes after two days of trametinib administration. Improvement in HCM was also noted [46].

### 3.2. Lymphatic Use of MEKi

Several clinical reports describe a successful use of trametinib to treat lymphatic fluid accumulation and the related secondary life-threatening cardiorespiratory, nutritional, and immune system complications in patients with various genotypes within the RASopathies. Nakano et al. treated three patients with NS due to pathogenic variants in *RIT1*, *SOS1* and *PTPN11,* aged 4 years, 3 and 4 months, respectively, with trametinib. They had primary central lymphatic dysplasia and life-threatening lymphatic accumulation affecting cardiorespiratory function, nutrition, and the immune system. Within one month of therapy, all three patients demonstrated a clinical response enabling weaning off of mechanical ventilation and other supportive care. Chest tube output volumes decreased, and enteral feeding could be advanced, resulting in improved nutritional status. Interestingly, one of these patients also had an NS-associated myeloproliferative disorder, which promptly responded to trametinib with a marked improvement in total white blood cells (WBCs) and absolute monocyte count: the reduction in WBC count was itself used as an initial indicator of efficacy in MEK inhibition after treatment start. The oral dose of trametinib administered initially ranged from 0.023 to 0.026 mg/kg/day and in two of the cases was subsequently reduced to 0.013 and 0.018 mg/kg/day due to gastrointestinal and skin AEs [26].

Dori et al. described an infant with *SOS1*-related NS with lymphatic disease who had significant improvement in retrograde lymphatic flow and duodenal lymphatic leaks after the initiation of trametinib and complete symptoms remission 8 weeks after treatment inception. In this case, the oral dose was progressively increased from 0.01 mg/kg/day to 0.02 mg/kg/day, with no AEs reported [39].

Li et al. reported a 13 year-old patient affected by advanced lymphatic disease due to *ARAF* mutation. Despite being unresponsive to conventional therapy and sirolimus, the boy showed dramatic improvements in chylotorax and pulmonary function tests after 2 months of therapy. Lymphatic remodeling with restructuring of his lymphatic system was evident at MRI. No AEs were reported. Interestingly, a preclinical test on a homologous zebrafish model was used to assess the efficacy of the drug in rescuing the phenotype [44].

Hribernik et al. presented a 3-year-old girl with an *RIT1* variant and NS, HCM, and refractory chylothorax who developed life-threatening chyle loss-related complications. Lymphatic remodeling with complete resolution of chylothorax was achieved by MEK inhibition with trametinib [42].

CCLA was successfully treated by Gordon and colleagues in a 22-year-old patient with NS due to an *RIT1* variant with widespread lymphatic resorption failure manifesting with ascites, pleural effusions, pericardial effusion, and subsequent cardiac tamponade. Trametinib resulted in prompt and sustained clinical improvement with complete resorption of ascites and pericardial effusion at 3 and 18 months of treatment, respectively. A general health improvement was noted, consistent with an increase in activity levels allowing a return to work. The reported AEs were nausea, gastritis, constipation, and eczema of the lower legs. The patient developed iron deficiency anemia requiring an iron infusion after 10 months on trametinib. An increase in trametinib dose to 0.0375 mg/kg/d exacerbated the eczematous skin lesions [41].

Two case reports showed promising results in treating KLA: Foster et reported an 18 year-old patient with KLA and pleural effusion due to a *CBL* variant who was effectively treated with 0.01 mg/kg/day 28-day cycles of trametinib, with complete symptom resolution and an improvement in the effusion. First improvements were seen after 1 week of therapy. The AEs included acneiform rash, transitory eosinophilia, and transient hematuria and proteinuria. Chowers and colleagues reported a 9-year-old patient with KLA and somatic *NRAS* pathogenic variant with recurrent pericardial and pleural chylous effusions showing improvement shortly after trametinib administration with an oral dose of 0.025 to 0.030 mg/kg/day. The AEs consisted of facial acneiform rash and hair loss [48].

Chakraborty and colleagues treated a 15-year-old boy with *PTPN11*-related NS, heart transplant, and mitral valve replacement presenting with diffuse massive hemorrhages involving lungs, nasopharynx, gastrointestinal system, and peritoneum that required artery embolization. Propranolol treatment was unsuccessful, while interferon 2b demonstrated efficacy but severe neutropenia required cessation of therapy. As complete coagulation and platelet assessments were within the normal range, hemorrhages were attributed to neo-angiogenesis phenomena. To control bleeding and inhibit neo-angiogenesis, 6.7 mg/m^2^ b.i.d oral selumetinib was administered, leading to hemorrhage stoppage. This allowed the patient to be discharged from the hospital without any further bleeding episodes in the year of observation. No adverse events were reported [35].

### 3.3. Neurological Use of MEKi

D’Onofrio et al. reported two patients with drug-resistant epilepsy treated with trametinib. The first, a 7-year-old patient affected by CFCS due to a *BRAF* pathogenic variant, experienced a relevant improvement in EEG anomalies after three months of trametinib at 0.025 mg/kg/day. There was also clinical improvement, and no new episodes of seizures occurred during the six-month observation period. In the second patient, a 26-month-old toddler affected by Schimmelpenning syndrome due to a *KRAS* mosaic variant, treatment with trametinib led to early benefits, with a seizure-free period of three months. Unfortunately, there was a significant rebound effect after discontinuation of the treatment due to suspected trametinib-induced inflammatory colitis [38]. It has been found that selumetinib can be useful for treating other aspects of NF1 apart from PN, as it has shown that it can potentially enhance executive function and working memory in children and young adults with NF1, while posing no significant neurotoxicity risks [60].

### 3.4. MEKi in Mosaic RASopathies

In 2021, Chang et al. reported on the first case of mosaic RASopathies treated with trametinib. They described a 15-year-old female with a *KRAS* pathogenic variant with an expanding high-flow facial arteriovenous malformation (AVM) that caused transfusion-dependent epistaxis. Despite multiple interventional procedures and after experiencing no success under sirolimus, the AVM worsened, along with the development of anemia. Trametinib administration led to the fading of her facial capillary malformation and no recurrence of epistaxis, consistent with AVM improvement at angio-MRI. The patient experienced some dermatological AEs, such as acneiform eruptions and intermittent paronychia, but they responded well to skin-directed therapies [36]. Nicholson et al. also described a case of AVM in a patient with a pathogenic variant in *EPHB4*. Trametinib was initiated as cardiac compromise developed. After 10 months of therapy consisting of thirteen cycles of 28 days, cardiac catheterization showed improvement in shunting, while the patient remained clinically stable with mild symptoms and a stable leg length discrepancy. However, the patient experienced skin and hair lightening, which was managed by reducing the trametinib dose from 2.0 mg daily to 0.5 mg daily [59].

Furthermore, a 13-month-old patient affected by congenital nevus melanocytic syndrome and leptomeningeal malformation and hydrocephalus secondary to cerebral malformation connected with a mosaic pathogenic *NRAS* variant was treated with trametinib, resulting in a dramatic but transient improvement in seizure frequency and weaning off antiepileptic medication. However, after 10 weeks of treatment, seizure activity increased in the absence of progression of the intracranial lesions, requiring the reintroduction and escalation of antiepileptic medication [37].

In 2022, Carli et al. reported a case of cutaneous skeletal hypophosphatemia syndrome (CSHS) caused by a mosaic *NRAS* variant successfully treated with trametinib. The patient, affected by a life-threatening hypophosphatemia unresponsive to conventional therapies, showed prompt and stable normalization of blood phosphate from day 11 of treatment. As the serum levels of calcium and phosphate ameliorated, the rickets score and cutaneous lesions also improved and catch-up growth occurred. Under treatment, this patient also showed a reduction in the thickness of an ameloblastoma of the scalp and a complete resorption of his chylothorax. The AEs experienced were asymptomatic hyperkalemia, dry skin, thick hair, and a transient elevation of serum CPK. We report here a follow-up of this case with respect to the original article. After treatment for 2.5 years, trametinib was withdrawn as no chylous effusion neither hypophosphatemia had occurred since treatment start. In the subsequent 6 months, phosphate urinary losses showed a slow upward trend and 7 months after he was admitted for severe recurrence of chylothorax requiring pleurodesis and thoracic duct ligation, unresponsive to octreotide and dietary modifications. Treatment with trametinib was then restarted and chylothorax was controlled after 20 days.

### 3.5. Indications, Dosage, Timing and Adverse Events

Treatment indications—Cardiological conditions, such as HCM and MAT, and lymphatic effusion caused by CCLA, KLA, or lymphatic dysplasia were the primary indications for treatment. Other indications included refractory bleeding (both internal and external), seizures, and MAT. Additionally, hypophosphatemic rickets was also an indication for treatment.

Dose—The recommended dose of trametinib for adult cancer treatment is 2 mg/day, which translates to 0.025 mg/kg/day for an 80 kg adult. For children with refractory solid tumors, a phase I study recommends a dose of 0.025 mg/kg/day for those aged six years or older and 0.032 mg/kg/day for those under six years [61]. In line with this indication, in most patients trametinib was administered orally as tablet, tablet crushed and resuspended, or as a liquid suspension from reconstituted powder, with a final dose of 0.025–0.032 mg/kg/day and a median dose of 0.030 mg/kg/day. The most frequent dose administered was 0.025 mg/kg/day. Few literature reports describe the use of escalating doses of trametinib [33,36,39,41,43], while in five cases therapy was de-escalated, aiming at mitigating adverse events that occurred during administration [26,38,46,47,59].

Treatment timing—There was a high variability in the first administration of MEKi, with treatment starting anywhere from 47 days to 22 years. Additionally, the time it took for patients to notice the first improvement after treatment was also highly variable, ranging from a few days to a few months. First improvements were reported after a variable length of time, spanning from days to 3 months, but it is worth noticing that the evaluated features were highly variable among different cases and also highly heterogeneous, depending on the clinical indication and the parameter monitored. The duration of therapy was not homogeneous among reports and clearly stated in only few of them, making it difficult to draw conclusions on long term efficacy. The maximum observation period reported was 2 years. In one case, despite clinical and electroencephalographic (EEG) improvements after trametinib initiation, regression of benefits with a relapse of symptoms occurred after few months of therapy administration [37]. Therefore, a transient effect cannot be ruled out.

Adverse events (AEs)—AEs occurred in nearly half of the patients (12 out of 23). Most of the AEs affected skin (nine cases) or the gastrointestinal system (four cases) and in some cases involved more than one system (four cases). Therapy was discontinued in two cases due to rhabdomyolysis [33] or cutaneous and gastrointestinal AEs [38]. Relapse of symptoms has been reported following stop treatment in two cases [34,38]

### 3.6. Monitoring Protocol

There are currently no shared recommendations for monitoring the potential toxicity of MEKi use in RASopathies. However, some authors have offered their approach regarding trametinib monitoring approaches, mainly based on its usage in adults with cancer [26,41,42,45,46,48,58,59] This approach involves periodic physical, skin and eye exams, ECG, echocardiography, chest radiography, MRI or CT (chest/abdominal), and blood tests to monitor for known side effects based on adult and pediatric clinical trials. These tests assess hematology, renal, liver, pancreatic and thyroidal function, coagulation, and BNP/NT-proBNP. The monitoring frequency can range from weekly to monthly to every 3 months, depending on the time since treatment start. Surveillance is aimed at identifying known dermatologic, pulmonary, gastrointestinal, hematologic, muscular, cardiovascular, and ophthalmologic side effects. In terms of biochemical monitoring, RNAseq has also been proposed as a research approach that could identify transcriptional modifications under treatment and help in linking biological changes with clinical improvement, as well as establishing an optimal dose related to side effects [47,58].

## 4. Discussion

While selumetinib was approved for treating children with NF1 with symptomatic, inoperable plexiform neurofibromas, other MEKis have not been systematically tested for treating other RASopathies [62]. A consensus on MEKi use in a variety of NF1-related manifestations, including tumor and non-tumor manifestations, was published [63]. Nevertheless, it is clear that the implementation of MEKi therapy in RASopathy requires more information about the efficacy and safety of these drugs for this specific indication. While compassionate/nominal use may be a therapeutic option for selected patients with a severe phenotype without better therapeutic options available, this approach is not applicable to the general RASopathy population. With this view, clinical trials are necessary to explore the potential clinical benefits of MEKi for RASopathies. However, designing trials for individuals with RASopathies is challenging due to several factors: the rarity of such conditions, their genetic and phenotypic heterogeneity, the variety of complications indicating treatment in these diseases, a limited knowledge of their natural history, the absence of validated therapeutic endpoints, and the ethical concerns preventing the definition of a control group under such circumstances. The economic aspect of developing drugs for rare diseases is also important, as clinical trials are costly and time-consuming. However, repurposing existing drugs could save both time and money. It is important to consider that the timely use of targeted therapy could lead to early benefits in the clinical course of the disease, ultimately reducing the impact on the healthcare system in terms of surgical procedures, hospitalizations, and complications. A phase 2 trial examining the safety, tolerability, pharmacokinetics, and pharmacodynamics of the MEKi binimetinib in individuals aged between 18 to 65 years old with NS HCM was withdrawn due to scientific and business considerations (NCT01556568). The MEKi trametinib showed promising results in treating various oncological conditions and preclinical studies on RASopathy models were more than encouraging. Although data on the efficacy and safety of trametinib are still limited, in certain cases of RASopathy, the severity of the disease and the absence of alternative treatments have necessitated its prescription under compassionate use, with the aim of ameliorating or halting disease progression.

In recent years, the medical literature has been enriched with promising reports detailing such experiences. Despite the heterogeneity across genotypes, nosology, age, dose, duration of therapy, and measured outcomes in these descriptions, nearly all patients experienced notable improvements in their conditions shortly after commencing treatment, often maintaining such improvements over an extended period. Furthermore, beyond addressing the primary indication for which the therapy was initiated, trametinib has demonstrated beneficial effects in other systems, frequently resulting in a comprehensive improvement in the diverse manifestations of RASopathies, such as catch-up growth. Thus far, the efficacy of MEK inhibitors (MEKis) has been empirically proved in reports of cases with variants in *RIT1*, *RAF1*, *SOS1*, *PTPN11*, *ARAF*, *KRAS*, *CBL*, *BRAF*, *EPHB4*, and *NRAS*. It is noteworthy that, due to its molecular mechanism of action, MEKis were ineffective in certain NS genotypes, such as *LZTR1* (Sewduth et al., 2020), and were not effective in treating patients with NS-ML associated with *PTPN11* mutations, as these missense changes have not been associated with increased MAPK signaling but signaling dysregulation through the PI3K-AKT-mTOR pathway (see below) [64].

However, despite these encouraging preliminary results and the enthusiastic tone of the authors, it is important to acknowledge the many limitations in the experiences reported so far. Firstly, the retrospective nature and potential publication bias of these case reports must be considered: treatment experiences are likely biased towards cases with severe and complicated phenotypes and the effectiveness of MEKis might not necessarily extend to cases with milder clinical presentations or less severe complications. Additionally, publication bias, which tends to favor reporting positive outcomes, could also skew the perceived effectiveness of trametinib, especially considering that there are likely more cases treated in clinical practice than those reported in the literature. Furthermore, trametinib has predominantly been studied in oncology, where the mutational spectrum of genes in the RAS-MAPK pathway differs from that of the RASopathies and the degree of hyperactivation of the pathway induced by oncogenic variants is stronger. Indeed, in oncology, an MEKi is typically administered in combination with other onco-suppressor therapies. However, in RASopathies, it may be reasonable to expect therapeutic benefits from single-agent MEKi therapy, as even a partial reduction in phospho-ERK1/2 activity could be sufficient. Secondly, the high heterogeneity among patients, genotypes, and conditions treated so far, as well as the diverse outcome measures defined by different authors, hinder a systematic evaluation of the treatment’s efficacy and preclude general indications beyond compassionate use.

In terms of AEs, trametinib use in pediatric oncology has shown that AEs are relatively common but mostly mild to moderate (grade 1–2), with skin and the gastrointestinal tract being most frequently affected. However, compared with reports in oncology, in the patients summarized here, AEs were less frequent than expected, and only two cases needed therapy discontinuation. This lower frequency of AEs may be attributable either to a different primary indication to treatment, different dose and treatment duration, and the absence of concomitant toxic anti-tumoral therapy, or rather be connected to the lack of a comprehensive and standardized monitoring protocol in the cases reported.

Furthermore, the long-term effects of trametinib therapy need further consideration, as the cases summarized here have a relatively short observation period, potentially presenting a flaw in detecting long-term efficacy and AEs. Indeed, while trametinib administration almost always resulted in a prompt clinical improvement, it did not prevent mortality in two cases, suggesting that its therapeutic effect may not address all pathological aspects of the disease, and transient responses have been documented.

On the other hand, trametinib treatment could also have a positive effect on other systems beyond the chief indication of the treatment. For instance, preliminary data from an ongoing multicenter phase II trial (NCT03363217) investigating the treatment of pediatric low-grade gliomas and plexiform neurofibroma showed a positive effect of trametinib on cognition in patients with NF1. Significant improvements in processing speed and visuo-motor and verbal abilities were observed.

Several questions still need to be investigated, including the optimal time window of treatment, drug dose and modifications over time, treatment duration and withdrawal, medium-to-long-term adverse effects, and relapses under therapy, as well as the impact on other manifestations within the RASopathies phenotype.

First, the outcomes of MEKis need to be compared with the natural history of the disease: Indeed, some children diagnosed with HCM who have received conventional treatment have shown spontaneous regression of ventricular wall thickness over time [65]. There is, therefore, a need for robust trials that take trametinib into consideration in complicated forms of RASopathy, despite the complexity and heterogeneity of its indications. Finally, an effort should be made to further document at a cellular and biochemical level the effects of trametinib: such experiments have been conducted in vitro on various mutant models to determine its effectiveness on a cellular level before being used to treat patients. Perhaps similar tests and ex vivo experiments could be of help in discriminating potential responders among patients before the drug is used in vivo. With this view, given the key role of the Ras-MAPK pathway, there is the need to adopt a monitoring strategy to understand the correlation between its inhibitions and the clinical outcome. Molecular biomarkers should be considered to assess the response in patients treated with trametinib.

Finally, drugs other than MEKis are currently under evaluation for their use in RASopathies for non-oncological indications: vosoritide to treat short stature (NCT04219007), simvastatin to inhibit growth and boned abnormalities in children with NS (NCT02713945), and lovastatin for improving synaptic plasticity, attention, and memory in NF1 patients (NCT03504501) [66].

Rapamycin, an mTOR inhibitor, has been shown to reverse HCM in an NS-ML mice model [64] but its administration in humans is anecdotal. A patient with *PTPN11*-related NS-ML experienced partial amelioration in HCM-related heart failure without reversal of cardiac hypertrophy [67]. Notably, a case of lymphatic malformation in keratinocytic epidermal nevus syndrome due to an activate *KRAS* variant partially responded to treatment with rapamycin [68]. It is important to note the absence of reports on the utilization of other Ras-MAPK inhibitors in the literature and their efficacy should also be considered for treating RASopathies.

In conclusion, the data summarized here strongly support the potential benefits of MEKis in managing the life-threatening complications of RASopathies. Drawing from our own experience and the findings outlined, we firmly advocate for considering MEKi treatment in cases of life-threatening events and complications in RASopathy patients. Furthermore, there is an urgent need for clinical trials to systematically assess the effectiveness and safety of MEKis in this context. Such trials are essential for advancing our understanding of and optimizing treatment strategies for individuals with RASopathies.

## Figures and Tables

**Table 1:** Clinical, genetic, and therapy characteristics of the reported cases treated with MEKi.

First Author and Year of Publication	Clinical Diagnosis	Genotype	Age	Drug	Dose	First Improvement	Chief Indication	Observation Period	Therapeutic Effect	Exitus	AE
Andelfinger, 2019 [34]	NS	*RIT1*:c.104G>C p.Ser35Thr, Heterozygous	14 weeks	Trametinib	0.020 mg/kg/day	3 months	HCM	17 months	HCM and NT-pro-BNP normalization. Valvular and subvalvular obstruction improved. Growth improvement.	-	-
Andelfinger, 2019 [34]	NS	*RIT1:*c.246T>G, p.Phe82Leu, Heterozygous	13 weeks	Trametinib	0.027 mg/kg/day	3 months	HCM	17 months	HCM and NT-pro-BNP normalization, valvular and subvalvular obstruction improved. Chylous effusions ceased. Growth improvement.	-	-
Li, 2019 [44]	GLA	*ARAF*:c.640T>C p.Ser214Pro, Heterozygous	13 years	Trametinib	1 mg/day	2 months	CCLA	1 year	Pulmonary function test improvement. Hyponatriemia and hypokaliemia resolution. Lymphatic remodeling with restructuring of lymphatic system.	-	-
Dori, 2020 [39]	NS	*SOS1*:c.2536G>A p.Glu846Lys, Heterozygous	5 years	Trametinib	0.010 mg/kg/day escalated to 0.02 mg/kg/day	-	Bleeding retroperitoneal and duodenal lymphangiectasia, chylothorax.	-	Symptom resolution. Improvement in chest radiograph signs. Normalization of albumin and hemoglobin levels without need for transfusion. Pulmonary edema and intestinal leakage reduction. Complete normalization of the duodenal mucosa.	-	-
Foster, 2020 [40]	KLA	*CBL*:c.2322T>G p.Tyr774*, VAF 4% frequency (pleural effusion fluid)	18 years	Trametinib	0.010 mg/kg/day in 28 day cycles, than daily	1 week	KLA with severe bilateral pulmonary interstitial and mediastinal perfusion	12 months	Subjectively improved symptoms. Improved restrictive lung disease. D-dimer value normalization. Complete resolution of pleural effusions and pulmonary interstitial edema.	-	Grade 1 acneiform rash, brief eosinophilia, and brief hematuria and proteinuria.
Meisner, 2021 [46]	NS	*RAF1*:c.770C>T p.Ser257Leu, Heterozygous	20 weeks	Trametinib	0.025 mg/kg/day de-escalated to 0.01875 mg/kg/day	2 days	MAT, HCM, and chylothorax	-	MAT episodes resolution. HCM improvement. Amiodarone and diltiazem weaning.	-	Severe diarrhea.
Chang, 2021 [36]	CM-AVM	*KRAS* p.Gln61His, low allele frequency consistent with mosaic origin (nasal epithelium)	15 years	Trametinib	uptitration to 1.5 mg/day	-	High-flow facial AVM with epistaxis	-	Clinical improvement and decreased epistaxis and need for RBC transfusion.	-	Acneiform eruptions and intermittent paronychia.
Nicholson, 2022 [59]	CM-AVM	*EPHB4*:c.2173G>A p.Ala725Thr (unspecified sample)	16 years	Trametinib	2 mg/day in 13 cycles of 28 days de escalated to 0.5 mg/day	-	Cardiac compromise in CM-AVM	-	Shunting improvement.	-	Acneiform eruptions, paronychia, air lightning.
Leegaard, 2022 [43]	NS	*RIT1*:c.170C>G p.Ala57Gly, Heterozygous	6 months	Trametinib	0.025 mg/kg/day escalated to 0.04 mg/kg/day	1 month	HCM	21 months	Complete regression of HCM	-	Dry skin and skin rash.
Mussa, 2022 [10]	NS	*RAF1*:c.770C>T p.Ser257Leu, Heterozygous	47 days	Trametinib	0.022 mg/kg/day	4 days	HCM	57 days	Weaning from mechanical ventilation, inotrope demand reduction, NT-proBNP values reduction, Ross score improvement, echocardiographic improvement with septal thickness reduction.	Refractory congestive heart failure due to pulmonary hypertension and pulmonary artery dilatation.	-
Lioncino, 2022 [45]	NS	*SOS1*:c.1655G>C p.Arg552Thr, Heterozygous	9 weeks	Trametinib	0.020 mg/kg/day	4 days	MAT and pleural effusion	4 months	Pleural effusion resolution, incessant MAT episodes resolution with anti-arrhythmic withdrawn, improvement in respiratory parameters with ventilation weaning, significant reduction in consolidation areas.	-	-
Nakano, 2022 [26]	NS	*RIT1*:c.246T>G p.Phe82Leu, Heterozygous	4 years	Trametinib	0.025 mg/kg/day	1 month	Chylotorax and HCM	2 years (administration time 1 year)	Chylotorax reduction, slight decline in NT-pro-BNP value. Growth improvement.	-	-
Nakano, 2022 [26]	NS	*SOS1*:c.1322G>A p.Cys441Tyr, Heterozygous	3 months	Trametinib	0.026 mg/kg/day de-escalated to 0.013 mg/kg/day	1 month	Chilotorax and ascites	2 y (administration time 1 year)	Chylotorax reduction, ventilation weaning, gradual improvement in pulmonary valve stenosis. Growth improvement.	-	Grade 2 skin irritation.
Nakano, 2022 [26]	NS	*PTPN11*:c.854T>C p.Phe285Ser, Heterozygous	4 months	Trametinib	0.023 mg/kg/day	1 month	HCM and chylotorax	few months	Chylous effusions and ascites improvement. Cardiac function stabilization, NT-proBNP reduction, HCM partial improvement, slight improvement in the pulmonary valve gradient. Mechanical ventilation weaning.	Yes. Acute cardiorespiratory event. The suspected cause was a sudden cardiac event.	-
Chakraborty, 2022 [35]	NS	*PTPN11*(unspecified) p.Gln510Glu	15 years	Selumetinib	6.7 mg/m^2^ twice a day	-	Refractory pulmonary and gastrointestinal bleeding	1 year	Bleeding resolution.	-	-
Hribernik, 2022 [42]	NS	*RIT1* variant (unspecified), Heterozygous	3 years	Trametinib	0.032 mg/kg/day	-	Chylothorax and HCM	8 months (Administration time 3 months)	Reduction in chest drainage frequency. HCM improvement.	-	Severe eczema and increased stoma output.
Gordon, 2022 [41]	NS	*RIT1*:c.246T>G p.Phe82Leu, Heterozygous	22 years	Trametinib	1 mg/day escalated to 2 mg/day	3 months	CCLA	28 months	Dramatic and sustained clinical improvement. He showed a total resolution of ascites within 3 months and pericardial effusion resolve 18 months into treatment	-	Mild nausea, gastritis, constipation, and eczema of the lower legs. Iron deficiency anemia requiring an iron infusion.
Carli, 2022 [47]	CSHS	*NRAS*:c.182A > G, p.Gln61Arg, VAF 29% (epidermal nevus)	4 years	Trametinib	0.032 mg/kg/day de-escalated to 0.025 mg/kg/day	11 days	Hypophosphatemic rickets and chylotorax	-	Normalization of serum phosphate and renal reabsorption., catch-up growth skin lesions, X-ray and densitometry bone mineralization improvement.	-	Hungry bone syndrome, asymptomatic hyperkaliemia and mild asymptomatic creatinine phosphokinase (CPK) elevation.
Sheppard, 2023 [33]	Oculoectodermal syndrome	*KRAS* p.Gly13Asp, VAF 10.3–38.8% (hyperpigmented skin, periosteum, muscle, and humerus samples)	-	Trametinib	0.025 mg/kg/day	-	CCLA	-	-	-	-
Sheppard, 2023 [33]	Mosaic KRAS-opathy	*KRAS*:c.436G > T p.Ala146Thr, VAF at 3.3–3.4% (scrotal tissue)	-	Trametinib	0.005 mg/kg/day	-	CCLA	-	-	-	Rhabdomyolysis
Chowers, 2023 [48]	KLA	*NRAS*:c.182A>G, p.Gln61Arg, VAF 0.2% (unspecified sample)	9 years	Trametinib	0.025 mg/kg/day	-	Pericardial and pleural chylous effusions	11 months	Symptoms and signs improvement, increased Lansky performance test, increased body weight, improvement in lung function tests, radiological improvement, wean off nocturnal oxygen supplementation, platelets count normalization and D-dimer decrease, weaning of steroid therapy.	-	Mild facial acneiform rash and hair loss.
D’onofrio, 2023 [38]	CFC	*BRAF* p.Phe595Leu, Heterozygous	7 years	Trametinib	0.025 mg/kg/day	3 months	Drug-resistant, recurrent tonic-colonic seizures	6 months	Dramatic reduction in electroencephalographic anomalies, marked clinical improvement.	-	-
D’onofrio, 2023 [38]	Schimmelpenning syndrome	*KRAS* p.Gly12Asp, postzygotic somatic (skin)	26 months	Trametinib	0.025 mg/kg/day escalated to 0.0375 mg/kg/day de-escalated to 0.025 mg/kg/day	-	Right focal motor seizures	22 months	Seizure frequency and skin lesions dimension reduction.	-	Suspected drug induced inflammatory colitis and hypovolemic shock during viral gastroenteritis. Eczematous skin lesions.
Copland, 2023 [37]	Congenital melanocytic naevus syndrome	*NRAS* Gln61Lys (skin)	13 months	Trametinib	0.032 mg/kg/die	28 days	Focal seizures	1 year	Transient seizure activity resolution, antiepileptic weaning, progression of motor skills, reduction in nevus pigmentation intensity. Progression of symptoms after 10 weeks of therapy.	-	-

Legend: AE, adverse event; NS, Noonan syndrome; HCM, hypertrophic cardiomyopathy; MAT, multifocal atrial tachycardia; GLA, generalized lymphatic anomaly; VAF, variant allele frequency; KLA, Kaposiform lymphangiomatosis; CFC, cardiofaciocutaneous syndrome; CCLA, central conducting lymphatic anomaly; CM-AVM: capillary malformation–arteriovenous malformation; CSHS: cutaneous skeletal hypophosphatemia syndrome; “-”, information not reported [26,33,34,35,36,37,38,39,40,41,42,43,44,45,46,47,48].
